# Mild Cognitive Impairment in Chronic Brain Injury Associated with Serum Anti-AP3B2 Autoantibodies: Report and Literature Review

**DOI:** 10.3390/brainsci11091208

**Published:** 2021-09-14

**Authors:** Niels Hansen, Dirk Fitzner, Winfried Stöcker, Jens Wiltfang, Claudia Bartels

**Affiliations:** 1 Department of Psychiatry and Psychotherapy, University Medical Center Göttingen, Von-Siebold-Str. 5, 37075 Goettingen, Germany; jens.wiltfang@med.uni-goettingen.de (J.W.); claudia.bartels@med.uni-goettingen.de (C.B.); 2 Department of Neurology, University Medical Center Göttingen, Robert-Koch Str. 40, 37075 Goettingen, Germany; dirk.fitzner@med.uni-goettingen.de; 3 Euroimmun Reference Laboratory, Seekamp 31, 23650 Luebeck, Germany; w.stoecker@euroimmun.de; 4 German Center for Neurodegenerative Diseases (DZNE), Von-Siebold-Str. 3a, 37075 Goettingen, Germany; 5 Neurosciences and Signaling Group, Institute of Biomedicine (iBiMED), Department of Medical Sciences, University of Aveiro, 3810-193 Aveiro, Portugal

**Keywords:** autoantibody, anti-AP3B2 antibody, cognitive impairment, autoimmunity, memory, traumatic brain injury

## Abstract

Background: Chronic traumatic brain injury is a condition that predisposes the brain to activate B-cells and produce neural autoantibodies. Anti-adaptor protein 3, subunit B2 (AP3B2) autoantibodies have thus far been associated with diseases affecting the cerebellum or vestibulocerebellum. Through this case report, we aim to broaden the spectrum of anti-AP3B2-associated disease. Case description: We report on a 51-year-old woman with a brain injury approximately 28 years ago who recently underwent neuropsychological testing, magnetic resonance imaging of the brain (cMRI), and cerebrospinal fluid (CSF) analysis. Neural autoantibodies were determined in serum and CSF. Our patient suffered from mild cognitive impairment (amnestic MCI, multiple domains) with stable memory deficits and a decline in verbal fluency and processing speed within a two-year interval after the first presentation in our memory clinic. Brain MRI showed brain damage in the right temporoparietal, frontolateral region and thalamus, as well as in the left posterior border of the capsula interna and white matter in the frontal region. Since the brain damage, she suffered paresis of the upper extremities on the left side and lower extremities on the right side as well as gait disturbance. Our search for autoantibodies revealed anti-AP3B2 autoantibodies in serum. Conclusions: Our report expands the spectrum of symptoms to mild cognitive impairment in addition to a gait disturbance associated with anti-AP3B2 autoantibodies. Furthermore, it is conceivable that a prior traumatic brain injury could initiate the development of anti-AP3B2-antibody-associated brain autoimmunity, reported here for the first time.

## 1. Background

Neural autoantibodies in psychiatric [1] and neurological disease [2,3,4] are having a growing impact on diagnosis and therapy. Developments in recent decades have led to discoveries of novel disease entities such as NMDAR encephalitis [5], limbic encephalitis [6], or autoimmune psychosis [7]. Neural autantibodies target glial and neuronal antigens and have various consequences often due to their location and distribution within the brain. Brain injury can trigger the production of neural autoantibodies (Table 1). Traumatic brain injuries (TBI) in particular vary in their causes, severity, and long-term outcomes in terms of behavior and cognition [8]. The immune system’s role and its targets in TBI remain unclear. It is known that TBI predisposes the brain to induce an immune reaction entailing lymphocyte infiltration and B-cell activation leading to the production of antibodies for neural antigens such as those described in patients that develop glial fibrillary acid protein autoantibodies [9,10] (Table 1). A paradigmatic example for autoimmunity developing after chronic TBI is the proof of antipituitary and antihypothalamus autoantibodies in patients suffering TBI-generated damage to the pituitary [11]. Various neural autoantibodies have been reported in conjunction with traumatic brain injury, i.e., anti-serotonin 2A receptor [12] or immunogenic proteins such as isoform Ib of synapsin 1 [13]. Autoantibodies against the adaptor protein 3, subunit B2 (AP3B2) have not been reported so far in association with chronic TBI. AP3B2 autoimmunity has been described in 10 patients with gait disturbance [14] and in those with cerebellar ataxia [15] and vestibulocerebellar syndromes [16]. Here we report the novel phenotype of recent progressing mild cognitive impairment associated with adaptor protein 3, subunit B2 (AP3B2) immunoglobulins in a female who experienced a chronic TBI.

## 2. Case Description

This 51-year-old business woman, married and mother of two children, presented in our memory clinic complaining of predominant and progressive memory disturbances. She initially came to our memory clinic two years ago with a cognitive impairment she believed had been getting even worse within the last years. She also reported a known but slightly worse gait disturbance. The gait disturbance originated from a head trauma followed by a coma lasting 10 days (caused by a car accident nearly 28 years earlier). Her coma involving consecutively diagnosed disorders affecting her memory, concentration, word-finding, and reading capabilities was the consequence of head trauma. Her cMRI shows a posttraumatic right temporoparietal, right frontolateral brain lesion in the thalamus and on the left posterior border of the capsula interna, as well as in left white matter in the frontal region. She developed structural epilepsy entailing secondary generalized seizures. Under treatment with lamotrigine (200 mg/d), she is currently seizure-free. Other comorbidities in her history are ferritin anemia, restless legs syndrome, and alcohol abuse previously (currently she is abstinent). She also suffers from neurodermitis and bronchial asthma. Her family anamnesis is inconspicuous concerning psychiatric or neurologic diseases. Psychopathological assessments revealed fluctuating mood and drive. Her depressive symptoms have been treated with citalopram (20 mg/d) and psychotherapeutical interventions. Neurological examination confirmed the known spastic paresis of the upper left-sided and lower right-sided extremities and a gait disturbance with upright stand and gait ataxia since her brain trauma. Furthermore, she exhibited bradydysdiadochokinesis and ataxic upper extremities, as well as bilateral square werve jerks.

Cognitive screening with the Mini-Mental Status Examination (MMSE) and Clock Drawing Test (CDT) revealed normal results, but extensive neuropsychological testing was indicative of a mild cognitive impairment (amnestic MCI, multiple domains) with deficits in verbal fluency, processing speed, flexibility, verbal und figural memory (Table 2).

In comparison to neuropsychological testing in 2018, deficient memory performance remained stable over time without further deterioration, but impairments in verbal fluency and processing speed declined slightly. Concurrently, her depressive symptoms increased and might have caused or contributed to the mild worsening of attentional and executive dysfunctions. A lumbar puncture to enable a differential diagnosis had been declined two years ago but was consented to at her last visit in 2020. This lumbar puncture aimed to determine specific autoantibodies, as she had begun to reveal progressive memory impairment a few years ago and a “red flag”, i.e., the gait disturbance and slight cerebellar ataxia. The autoantibodies we analyzed were: via antibody blots [antibodies against intracellular antigens: Amphiphysin, CV2, HuD, glutamic acid decarboxylase (GAD65), SOX1, Ma1/2, Ri, Ro, TR, Yov and Zic4] and immunofluorescence tests [antibodies against cell-surface antigens: α-amino-3-hydroxy-5-methyl-4-isoxazolepropionic acid receptor 1/2 (AMPAR1/2), Aquaporin 4, contactin associated protein 2 (CASPR2), dipeptidyl-peptidase–like protein-6 (DPPX), gamma-aminobutyric acid B1/ 2 (GABAB1/2), leucin rich glioma inactivated protein 1 (LGI1), NR1 subunit of the N-methyl-D-aspartate receptor (NMDAR) ]. Using fluroescence brain-tissue slides, we set a 1:10 intensity as the cut-off for positivity compared to the control slides. CSF testing revealed no destruction markers and no pleocytosis. Serum cell-based antibody analysis showed anti-AP3B2 antibodies with an intensity of 1:100 (Table 2). 1:100 was set as the cut-off for anti-AP3B2 antibodies positivity. No molecular neurodegeneration was diagnosed, as her molecular markers in CSF were within the normal range (Table 2). We assumed an autoimmune origin of MCI and started immunosuppressive therapy with high-dose intravenously applied corticosteroids. Consecutive neuropsychological testing after the first cycle of corticosteroids revealed improved semantic word fluency, cognitive flexibility, and consolidation of verbal, non-associated information. Fluor-18 FDG PET revealed no hint of cancer throughout her body. Brain Fluor-18 FDG PET revealed photopenia on the right parietal side, concurring with her condition after the brain trauma.

## 3. Discussion and Conclusions

Our case report highlights both the importance of a prior brain lesion that might predispose the brain to develop an autoimmune process and enlarges the clinical spectrum of AP3B2 antibodies. AP3A is a protein in nerve cell bodies that is important for the transport of proteins by regulating endosomes to synaptic vesicles, and for forming synaptic vesicles [23,24]. Autoantibodies against AP3B2 damage this transport machinery, impeding synaptic vesicle formation and incurring consecutive deficits within the synaptic function. Bearing this AP3B2 function in mind, it is conceivable that our patient’s cognitive impairment is being caused by synaptic dysfunction triggered by AP3B2 autoantibodies. Furthermore, it is probable that her prior brain damage involving consecutively activated B-cells and antibody production (evident in spinal cord injury in mice [25] could result in a brain inflammation entailing neuronal cell death. 

A study by Goryunova et al. [19] in children showed that neural autoantibodies against membrane surface receptors such as NMDAR or AMPAR might be upregulated in children who suffer a chronic TBI. The chronic TBI might also cause a disruption in the blood-brain barrier leading to brain inflammation and dementia, as proposed by Abrahamson and Ikonomovic [26] Other types of brain injury such as ischemia are known to be accompanied by antibodies against the NMDAR subunit NR1 that are closely associated with the outcome of neuropsychiatric symptoms [27]. The NMDAR, in particular, might play an important role in TBI, as it has an important function in regulating synaptic plasticity and memory formation [28] explaining predominant learning and memory deficits in patients with NMDAR antibodies, as a study by Datta et al. showed [29]. The role of AP3B2 antibodies is less clear than those of the NMDAR antibodies. AP3B2 autoantibodies might have accessed the peripheral blood via the leaky blood-brain barrier. Some researchers have hypothesized that serum autoantibodies to different brain antigens in TBI’s posttraumatic phase reflect the damage occurring in antigenic structures in the blood-brain barrier resembling different membrane receptors of neurofunctional proteins [3,4]. The expression and distribution of these membrane receptors antibodies might hold important clues for the neuropsychiatric symptoms we observed. However, neural autoantibodies do not always play a role in inducing neuropsychiatric dysfunction—they can function protectively in glutamate excitotoxicity in mild TBI in children, such as the antibody against the NR2 subunit of the NMDAR as Sorokina et al. hypothesized in their study [30]. 

However, antibodies against another NMDAR subunit, namely NR1, are known to be associated with ischemic lesions with the occurrence of neuropsychiatric symptoms entailing cognitive decline, as mentioned above [27]. Autoantibody-associated TBI can be accompanied by substantial neurodegeneration [31]. However, it appears that there is no simultaneous neurodegeneration occurring in addition to the autoimmunity involving the production of AP3B2 autoantibodies, as we detected no abnormal ranges of markers of unspecific axonal neurodegeneration and beta-amyloid. Undetected amyloid-based neurodegeneration is a good prognostic factor, as we know that a substantial proportion of TBI patients present ß-amyloid plaques—a risk factor for Alzheimer’s disease [32] occurring early in TBI’s posttraumatic phase [33]. The cognitive decline before the application of corticosteroids argues against the traumatic brain injury as the major cause of the progression as it is more likely that the traumatic brain injury 28 years ago causes a more stable, not further deteriorating cognitive impairment. Thus, it is more likely that cognitive impairment is possibly related to AP3B2 autoimmunity although we do not know when does autoantibodies first appear as these have not been tested earlier. Furthermore, cognitive deficits with AP3B2 autoimmunity seem to be susceptible to treatment with corticosteroids. 

The depressive syndrome only slightly worsened in the last years so that it cannot be excluded that depression might also be a manifestation of AP3B2 antibody-related immunity. However, it is also possible that the depressive symptoms have been stable in the long run and might be due to the postconcussive syndrome that developed after brain injury. However, it remains an open question whether traumatic brain injury triggers anti-AP3BS autoantibodies to result in a phenotype with cognitive dysfunction or whether these abnormalities are unrelated epiphenoma. Diagnostic lumbar puncture including neural autoantibody analysis in presence of additional hints for autoimmunity may help to clarify this and other autoantibodies—clinical phenotype associations. Furthermore, the increase in anti-AP3B2 antibodies in our patient’s serum after TBI, and her responsivity to immunosuppressive therapy argue for a link between these autoantibodies and damaged synaptic transmission processes.

In conclusion, we report here the rare case of a woman with a chronic brain injury that might have triggered an autoimmune process entailing the production of AP3B2 autoantibodies. However, we emphasize that these autoantibodies’ aforementioned association does not prove a causative relationship between TBI and AP3B2 antibodies. Our report expands the clinical spectrum of AP3B2 disease to a manifestation characterized by a predominant amnestic MCI in multiple cognitive domains. Furthermore, this is the first time that AP3B2 autoantibodies have been demonstrated in association with chronic traumatic brain injury. They may eventually prove to be a novel biomarker target in TBI warranting further investigation.

## Figures and Tables

**Table 1:** Serum neural autoantibodies associated with brain injury.

Autoantibody Directed against Antigen	References
Alpha 7 subunit of AchR	[17]
AMPAR	[18,19]
GFAP	[9,10]
Hypothalamus	[11,20]
NMDAR	[19,21]
Pituitary	[11,22]
Serotonin 2A receptor	[12]
SYN1	[13]

Abbreviations: AchR = acethlycholine receptor, AMPAR = α-amino-3-hydroxy-5-methyl- 4-isoxazolepropionic acid receptor, GFAP = glial fibrillary acid protein, NMDAR = N-methyl-D-aspartate receptor, SYN1 = isoform Ib of synapsin 1.

**Table 2:** Laboratory and clinical data at first presentation.

Laboratory Parameters	Data
AP3B2 Serum	1:100
AP3B2 CSF	negative
***Cells CSF***	
Cells/µL (˂5 µg/L)	0
Lymphocytes %	93
Monocytes %	6
Lactat mmol/L	1.4
***Proteins CSF***	
Albumin mg/L	219
IgG mg/L	32.5
IgA mg/L	2.5
IgM mg/L	0.38
QAlb %	5.1
QIgG %	2.6
QIgA %	1.1
QIgM %	0.31
***Destruction Marker Serum/CSF***	
Serum NSE (<30 ng/mL)	22.8
CSF NSE (<30 ng/mL)	14.7
Serum S100B (<0.15 µg/L)	0.09
CSF S100B (<2.7 µg/L)	2.3
CSF Tau protein pg/mL (<450 pg/mL)	<75
CSF P-Tau 181 protein pg/mL (<61 pg/mL)	34
CSF ß-Amyloid 1–42 pg/mL (>450 pg/mL)	1171
CSF ß-Amyloid 1–40 pg/mL	6667
CSF ß-Amyloid 1–42/–140 × 10 (>0.5)	1.8
**Neuropsychological and Clinical Parameters**	
BDI-II (depression) raw score	38
MMSE (screening) raw score	28
CDT (screening) raw score	01
CERAD Boston Naming Test (language) z-score	0.5
CERAD semantic fluency (language) z-score	−1.4
CERAD phonemic fluency (language) z-score	−2.0
WAIS−IV Coding (processing speed) z-score	−1.3
TMT Part A (processing speed) z-score	−2.8
TMT Part B (flexibility) z-score	−2.6
RWT semantic fluency—alternating (flexibility) z-score	−2.2
RWT letter fluency—alternating (flexibility) z-score	−2.0
CERAD Figure Copy (visuoconstruction) z-score	0.8
RCFT Copy (visual functions) z-score	≥−1.0
WAIS-IV Block Design (visuoconstruction) z-score	0.0
WAIS-IV Digit Span forward (memory span) z-score	0.0
WAIS-IV Digit Span backward (working memory) z-score	−0.3
CERAD List Learning 1–5 (verbal learning) z-score	−0.4
CERAD List Free Recall (verbal long-term memory) z-score	−1.6
CERAD List Savings (verbal long-term memory) z-score	−2.9
CERAD List Recognition (verbal long-term memory) z-score	−2.2
WMS-IV Logical Memory I (verbal immediate recall)	−1.7
WMS-IV Logical Memory II (verbal delayed recall) z-score	−3.0
WMS-IV Visual R I (visual immediate recall) z-score	−1.3
WMS-IV Visual R I (visual delayed recall) z-score	−2.0

For laboratory data normal ranges are given in brackets. For neuropsychological parameters z-values as normative data are presented whenever applicable. Z-values < −1 denote performance below the normal range, z-values ≥ −1 denote performance within the normal range. Abbreviations: Alb = albumin, BDI-II = Beck Depression Inventory, CERAD = Consortium to Establish a Registry for Alzheimer’s Disease, CDT = Clock Drawing Test, IgA = immunoglobulin A, IgG = immunoglobulin G, IgM = immunoglobulin M, MMSE = Mini Mental Status Examination, P-Tau 181 = phosphorylated tau protein 181, Q = quotient, Visual R I = Visual Reproduction I, RCFT = Rey Complex Figure Test, RWT = Regensburg Word Fluency Test, TMT = Trail Making Test, WAIS-IV = Wechsler Adult Intelligence Scale- fourth edition, WMS-IV = Wechsler Memory Scale ‒fourth edition.

## Data Availability

Data are available from the corresponding author.
